# High-yield ‘one-pot’ biosynthesis of raspberry ketone, a high-value fine chemical

**DOI:** 10.1093/synbio/ysab021

**Published:** 2021-08-20

**Authors:** Simon J Moore, Tommaso Tosi, David Bell, Yonek B Hleba, Karen M Polizzi, Paul S Freemont

**Affiliations:** Centre for Synthetic Biology and Innovation, Imperial College London, South Kensington Campus, London, UK; Department of Medicine, Imperial College London, South Kensington Campus, London, UK; School of Biosciences, University of Kent, Canterbury, UK; Department of Medicine, Imperial College London, South Kensington Campus, London, UK; Centre for Synthetic Biology and Innovation, Imperial College London, South Kensington Campus, London, UK; Department of Medicine, Imperial College London, South Kensington Campus, London, UK; Centre for Synthetic Biology and Innovation, Imperial College London, South Kensington Campus, London, UK; Department of Life Sciences, Imperial College London, South Kensington Campus, London, UK; Centre for Synthetic Biology and Innovation, Imperial College London, South Kensington Campus, London, UK; Department of Life Sciences, Imperial College London, South Kensington Campus, London, UK; Centre for Synthetic Biology and Innovation, Imperial College London, South Kensington Campus, London, UK; Department of Medicine, Imperial College London, South Kensington Campus, London, UK

**Keywords:** synthetic biology, fine chemical, raspberry ketone, polyketides, cell-free

## Abstract

Cell-free extract and purified enzyme-based systems provide an attractive solution to study biosynthetic strategies towards a range of chemicals. 4-(4-hydroxyphenyl)-butan-2-one, also known as raspberry ketone, is the major fragrance component of raspberry fruit and is used as a natural additive in the food and sports industry. Current industrial processing of the natural form of raspberry ketone involves chemical extraction from a yield of ∼1–4 mg kg^−1^ of fruit. Due to toxicity, microbial production provides only low yields of up to 5–100 mg L^−1^. Herein, we report an efficient cell-free strategy to probe into a synthetic enzyme pathway that converts either L-tyrosine or the precursor, 4-(4-hydroxyphenyl)-buten-2-one, into raspberry ketone at up to 100% conversion. As part of this strategy, it is essential to recycle inexpensive cofactors. Specifically, the final enzyme step in the pathway is catalyzed by raspberry ketone/zingerone synthase (RZS1), an NADPH-dependent double bond reductase. To relax cofactor specificity towards NADH, the preferred cofactor for cell-free biosynthesis, we identify a variant (G191D) with strong activity with NADH. We implement the RZS1 G191D variant within a ‘one-pot’ cell-free reaction to produce raspberry ketone at high-yield (61 mg L^−1^), which provides an alternative route to traditional microbial production. In conclusion, our cell-free strategy complements the growing interest in engineering synthetic enzyme cascades towards industrially relevant value-added chemicals.

## 1. Introduction

Cell-free systems offer an intriguing solution to reconstitute metabolic pathways without the genetic constraints of a living cell. The definitions of cell-free systems vary in the literature and include the use of pure enzymes (1–4) or cell extracts enriched with heterologous enzymes (5–8), while, alternatively, metabolic enzymes can also be generated *in situ* through cell-free protein synthesis (5, 9). These combined approaches present an alternative concept to traditional cell-based metabolic engineering, with the potential for efficient biosynthesis of complex natural products (7, 10, 11), screening of biosynthetic enzymes (5, 6, 12), optimizing cofactor regeneration schemes (1, 12, 13) or probing biosynthetic promiscuity (14). However, before cell-free technologies can be used for the synthesis of lower value-added products such as fine chemicals, there are several aspects that need to be addressed including cost, scalability and the longevity of the extract or purified enzymes. In this short communication, we focus on examining the feasibility of enzyme biosynthesis of 4-(4-hydroxyphenyl)butan-2-one—also referred to as raspberry ketone—a fine chemical from raspberries (*Rubeus rubrum*).

Raspberry ketone is harvested via a highly inefficient extraction from raspberry fruit, with an approximate yield of ∼1–4 mg per kg of fruit, which leads to a high market value of ∼$10 000–20 000 per kg and global production estimated at between 100 and 200 tons per annum (15). The natural raspberry ketone biosynthetic pathway is not fully resolved but is derived from L-tyrosine or phenylalanine and belongs to the plant flavonoid natural product family, which includes curcumin, naringenin, resveratrol and gingerole (16). To make raspberry ketone, a combination of plant, fungal and bacterial enzymes is be used, starting from *p*-coumarate (15). First, *p*-coumarate is activated for acyl chain extension by the addition of Coenzyme A (CoA) by an ATP-dependent CoA ligase (PCL) to form *p*-coumaroyl-CoA (17). Following this, the benzalacetone synthase (BAS) uses malonyl-CoA for acylation, which includes an unusual double decarboxylation event to cause the 4-(4-hydroxyphenyl)-buten-2-one intermediate, also referred to as 4-hydroxybenzalacetone (HBA) (18). Finally, an NADPH-dependent raspberry ketone/zingerone synthase (RZS1), a double bond reductase, reduces HBA into raspberry ketone (19). At present, despite its simplicity, raspberry ketone is a challenging molecule to make through microbial production. Key limitations include malonyl-CoA regeneration (20) and the general metabolic toxicity (through growth inhibition) of the raspberry ketone pathway intermediates (15). Therefore, current microbial fermentation strategies, starting from *p*-coumarate, have produced limited yields in either *E. coli* or *Saccharomyces cerevisiae*, ranging from 0.2–95 mg L^−1^ (15, 21–23), which largely rely upon growth media optimization and maximizing cell density to overpower general toxicity issues from ketone intermediates. To indirectly overcome growth inhibition, recently Yang *et al* used a *E. coli* whole-cell catalyst approach to make up to 9 g L^−1^ of raspberry ketone from HBA (24). However, this strategy bypasses the polyketide step and incubates *E. coli* cells containing a pre-expressed RZS1 and a glucose dehydrogenase for NADPH regeneration, in an optimized reaction buffer with HBA (24).

Herein, we establish an alternative ‘one-pot’ multienzyme approach for making raspberry ketone from L-tyrosine with cofactor regeneration, in order to study biosynthetic dexterity outside the constraints of a living cell. One of the essential considerations for cell-free biosynthesis is the cofactor costs. While a range of low-cost cofactor regeneration systems are available (25), the expense and chemical stability of specific cofactors such as NADPH is a limitation. For ‘one-pot’ raspberry ketone biosynthesis, optimal conversion was initially obtained with NADPH, while minimal yields were observed with NADH, due to unfavorable kinetic properties of RZS1 for NADPH. This is important since the bulk price per mole of NADH ($3000) is a fraction of NADPH ($215 000) (26). Therefore, to reduce the overall cost of cell-free raspberry ketone synthesis, a key focus of our current work was on relaxing the cofactor specificity of RKS1, which has previously been achieved for other reductases (1, 27). We do this by obtaining a stable ternary structure of RKS1 in complex with NADPH and HBA, which we then use to rationally engineer the cofactor preference of RKS1 towards NADH. Finally, we employ this new engineered RKS1 variant (G191D) to establish a multienzyme ‘one-pot’ raspberry ketone biosynthesis from inexpensive precursors.

## 2. Materials and methods

### **2.** **1** Molecular biology and protein expression

Raspberry ketone and cofactor regeneration genes (see supporting information) were synthesized by ThermoFisher Scientific and codon optimized for *E. coli* K12 expression with compatibility for EcoFlex cloning using routine methods (28). All oligonucleotides, plasmids and synthetic DNA sequences are listed in the supporting information (Tables S4 and S5). Sequencing was performed by Eurofins, Germany.

## 3. Golden gate mutagenesis

We developed a new protocol for mutagenesis based on Golden Gate cloning. Forward and reverse primers were designed for inverse polymerase chain reaction (PCR) of the plasmid (pET15b-RZS1) to incorporate a BsaI site. After digestion with BsaI, the restriction sites are removed, leaving a complementary 4 bp overhang, which is designed to incorporate the mutation site. This anneals and ligates to provide the desired mutation. PCR was performed with the Q5 polymerase (NEB) with 3% (v/v) dimethyl sulfoxide using the manufacturers standard guidelines. PCR was run on a 1% (w/v) agarose gel and the bands were excised and gel purified with a QIAquick Gel Extraction Kit. 20 ng of PCR product was then digest and ligated in a ‘one-pot’ reaction with 1× DNA ligase buffer (Promega), one unit of BsaI-HF, five units of T4 ligase and one unit of DpnI. The reaction was run for 15 cycles at 37°C for 5 min and 16°C for 10 min, followed by a further incubation at 50°C for 5 min and 80°C for 5 min. 20 μL of DH10β competent cells were transformed with 2 μL of ligation mix and plated onto 100 μg mL^−1^ carbenicillin plates. Single colonies were then sequence verified for incorporation of the mutation site.

### **3.** **1** Protein expression and purification

His_6_-tagged recombinant TAL, PCL, BAS, MatB and RZS1 were over-produced in *E. coli* BL21-Gold (DE3) grown at 37°C, 200 rpm in 2YT medium with 100 μg mL^−1^ ampicillin until an OD_600_ of 0.6 was reached. Cells were induced with 0.4 mM IPTG and grown overnight at 21°C at 200 rpm. Cell were collected by centrifugation at 6000 × *g*, 4°C for 20 min, and then re-suspended in binding buffer (20 mM Tris-HCl pH 8500 mM NaCl, 5 mM imidazole) and lysed by sonication. Cell-lysates were clarified with centrifugation at 45 000 × *g*, 4°C for 20 min and purified by gravity flow using Ni-NTA agarose (Generon). His_6_-tagged proteins were washed with increasing concentrations of imidazole (5, 30 and 70 mM) in 20 mM Tris-HCl pH 8500 mM NaCl, before elution at 400 mM imidazole. Purified proteins were then dialyzed (MWCO 10 000) into 2 l of 20 mM HEPES pH 7.5, 100 mM NaCl at 4°C for 6 h. The enzymes were found to be soluble and active in a range of standard buffers including HEPES (pH 7.5) and Tris-HCL pH 8.0–9.5. Additionally, all enzymes were stable for long-term storage at −80°C with 15% (v/v) glycerol.

### **3.** **2** Chemical synthesis of HBA

HBA, also referred to as 4-(4-hydroxyphenyl)-buten-2-one, was synthesized by a crossed aldol condensation as previously described (23, 29).

### **3.** **3** Liquid-chromatography mass spectrometry of raspberry ketone and pathway intermediates

50 μL samples of time-course reaction in triplicate were removed and inactivated with 450 μL of 1% (v/v) HCl. Samples were centrifuged at 21 000 × *g* for 10 min at room temperature. The supernatant was directly analyzed by liquid-chromatography mass spectrometry (LC-MS), performed with an Agilent 1290 Infinity system with an online diode array detector in combination with a Bruker 6500 quadruple time-of-flight (Q-ToF) mass spectrometer. An Agilent Extend-C18 2.1 × 50 mm (1.8 μm particle size) column was used at a temperature of 25°C with a buffer flow rate of 0.2 mL^−1^ min^−1^. LC was performed with a linear gradient of buffer A [0.1% (v/v) formic acid] and buffer B [0.1% (v/v) formic acid in acetonitrile]. Separation was achieved using 5% (v/v) buffer B for 2 min, followed by a linear gradient to 50% (v/v) buffer B from 2 to 9 min, which was held at 50% (v/v) buffer B from 9 to 10 min. Spectra were recorded between a mass range of 90–1000 *m/z* at a rate of 3 spectra per second. Standards were prepared and calibration curves for the intermediates L-tyrosine, *p*-coumaric acid, HBA and raspberry ketone were derived. Quantitation was based on the MS peak area of precursor or fragment ion in comparison to the analytical standards. Under the conditions used, raspberry ketone is detected as a sodium adduct [M + Na^+^]^+^ or as a diagnostic fragment ion at *m/z* = 107.49, corresponding to C_7_H_7_O. For the standards in solvent, good linearity (R^2^ > 0.99) was achieved over the range of 0.3–30 pmol on column. The lower limit of quantitation was set at 0.3 pmol. Samples that were below this limit were repeated by increasing the injection volume to 1 μL. Due to a lack of an analytical standard and poor separation, *p*-coumaroyl-CoA was not quantified.

### **3.** **4** Enzyme kinetics

RZS1 and mutants were purified to homogeneity using nickel IMAC and buffer exchanged into Buffer A. Steady-state kinetics were monitored on a Clariostar (BMG) plate-reader monitoring absorbance at 340 nm following the reduction of NAD(P)H to NAD(P)^+^ with either 4-hydroxyphenyl-3-butan-2-one (hydroxybenzaldehyde) or phenyl-3-butan-2-one (benzaldehyde) as substrates. Assays were performed in triplicate at 30°C in 0.1 M potassium phosphate pH 6.4.

### **3.** **5** RZS1 crystallization and structure determination

IMAC purified RZS1 was dialyzed for 4 h in 20 mM Tris-HCl pH 8.0 and 200 mM NaCl. Purified fractions were concentrated with a 10 000 MWCO centrifugation concentrator (Amicon) and then separated by analytical Superdex S200 gel filtration column (GE Healthcare) in the same buffer with a flow rate of 1 mL min^−1^. Purity was assessed by sodium dodecyl sulphate-polyacrylamide gel electrophoresis (SDS-PAGE), and the concentration was determined by A_280_ measurement using an extinction coefficient of 44 030 M^−1^ cm^−1^. RZS1 was concentrated to 10 mg mL^−1^ and screened in a range of crystallization conditions using 300 nL drops containing either a ratio of 1:2 or 2:1 of protein and reservoir buffer. Crystals of N-terminally His_6_-tagged RZS1 were obtained by sitting drop vapor diffusion at 20°C after ∼3 days of incubation in 0.1 M MES/imidazole pH 6.3, 11% (w/v) PEG 550 MME and 5% (w/v) PEG 20 K, 20 mM of amino acid mixture (Molecular dimensions Morpheus system) with 1 mM NADPH. Single cube-shaped crystals grew within 1 week. Native crystals with NADPH bound were soaked in cryoprotectant containing 1 mM HBA, 1 mM NADPH and 20% glycerol. A native dataset of 1800 frames was collected remotely at the I04 beamline (Diamond Light Source, Didcot, Oxfordshire) from a single crystal diffracting up to ∼1.5 Å. The crystal belonged to space group P1 (Table S1). Further details on structure determination are provided in the supporting information. The atomic coordinates and structure factors (codes: 6EOW for the ternary structure) have been deposited in the Protein Data Bank.

## 4. Results

### **4.** **1** ‘One-pot’ biosynthesis of raspberry ketone without cofactor recycling

**Figure 1.**
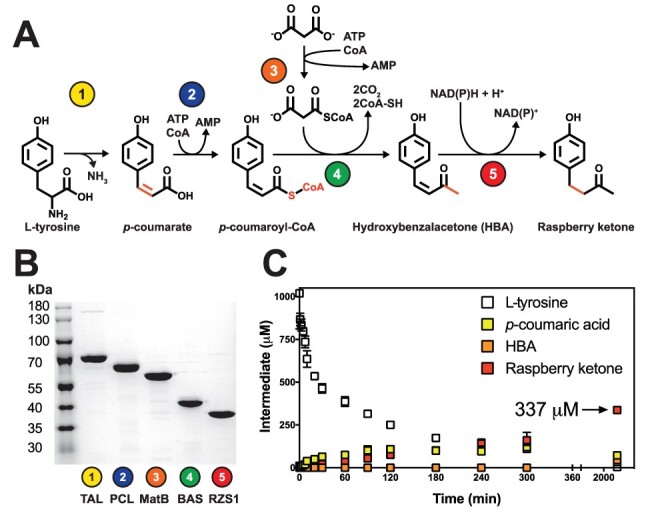
A cell-free module for raspberry ketone biosynthesis. A. A synthetic pathway for raspberry ketone synthesis from L-tyrosine using malonate for malonyl-CoA synthesis. B. SDS-PAGE of pathway enzymes. 2 μg of purified enzyme was loaded and analyzed by 12% (v/v) SDS-PAGE and Coomassie Blue staining. Sizes of His_6_-tagged recombinant enzyme—*R. glutinis* TAL (77.0 kDa), *A. thaliana* PCL (63.2 kDa), *R. palustris* MatB (56.6 kDa), *R. palmatum* BAS (44.4 kDa), *R. rubrum* RZS1 (40.7 kDa). C. ‘One-pot’ biosynthesis of raspberry ketone with 2.5 μM of enzymes under standard conditions. For full details see Table S2. Intermediates quantified by LC-MS include L-tyrosine (white box), *p*-coumarate (yellow box), HBA (orange box) and raspberry ketone (red box). Data are representative of three technical repeats.

In order to study the biosynthesis of raspberry ketone, we selected a synthetic five-step pathway for raspberry ketone using a combination of bacterial, fungi and plant enzymes (Figure 1A). Based on this synthetic pathway, a set of highly active and well-characterized enzymes. *Rhodotorula glutinis* TAL (30) and *Arabidopsis thaliana* PCL (17) were selected from the BRENDA database (see methods) to afford *p*-coumaroyl-CoA from L-tyrosine, along with the *Rhodopseudomonas palustris* MatB (31) for provision of malonyl-CoA from malonate, CoA and ATP. In addition, we also selected the *Rheum palmatum* BAS (18) and *Rubreus rubrum* RZS1 (19), where only a single enzyme has been reported for conversion of *p*-coumaroyl-CoA into raspberry ketone. Initially, each enzyme was recombinantly produced in *E. coli* BL21 Gold (DE3) pLysS and purified at high-yields (∼10–100 mg L^−1^) from the soluble fraction with at least 95% purity as estimated by SDS-PAGE (Figure 1B). The activity of TAL, PCL and RZS1 enzymes was also assayed individually (Figure S1), and results were in close agreement to previous reported literature values (17, 19, 30).

To begin, we tested a ‘one-pot’ reaction with 5 μM of purified TAL, PCL, MatB, BAS and RZS1 enzymes incubated with 1 mM L-tyrosine and substrates/cofactors (MgATP, CoA, malonate and NADPH). Please refer to the materials and methods for further reaction conditions. Samples were removed at time points and quantified by reverse phase (C18) LC-MS (Figure 1B). To begin, L-tyrosine is steadily depleted, while *p*-coumarate accumulates up to a maximum of 110 μM. Under these conditions, the HBA intermediate was not detectable, with the RZS1 fully converting its substrate into raspberry ketone. Raspberry ketone biosynthesis occurred at an initial linear rate of 0.63 μM min^−1^ for up to 6 h, with a 33.7% conversion (337 μM) achieved at end-point, which is equivalent to 55 mg L^−1^ raspberry ketone under batch synthesis. This suggests that the synthetic pathway was highly active, which was intriguing based on the relatively low-productivity observed in cell-based metabolic engineering efforts (from 0.28 mg L^−1^ and 95 mg L^−1^ in yeast and *E. coli*, respectively) (15, 21–23).

### **4.** **2** Cofactor engineering of the RZS1 enzyme for NADH preference

Next, we focused our attention on relaxing the NADPH specificity of the RZS1 enzyme. RZS1 catalysis the NADPH-dependent double bond reduction of HBA into raspberry ketone. For cell-free based biocatalysis, NADH or biomimetic analogues (32) are preferred to provide increased stability and reduced cost for reductive enzyme reactions. We therefore repeated the ‘one-pot’ biosynthesis of raspberry ketone with NADH in place of NADPH as a cofactor (Figures S2 and S3). In this reaction, the rate of L-tyrosine deamination by TAL remained constant, and an increased accumulation of *p*-coumarate (172 μM) and HBA (15 μM) is observed after 6 h, with only trace levels of raspberry ketone detected. After 48 h, a 7.6% (76.1 μM) conversion of raspberry ketone was achieved. The initial lag in raspberry ketone accumulation confirmed that the RZS1 enzyme had a clear preference for NADPH as a cofactor.

To further understand the RZS1 reductase, the enzyme was next characterized *in vitro*. First, HBA the natural substrate for RZS1 is yellow in coloration at pH >6 and strongly absorbs between 250 and 450 nm, which overlaps with NAD(P)H absorbance at 340 nm for kinetic characterization. Instead, to initially obtain kinetic parameters for the RZS1 enzyme, the substrate analogue phenylbuten-2-one was used since it lacks UV-Visible absorbance at 340 nm. To begin, the RZS1 enzyme shares sequence similarity (77% amino acid identity) and similar kinetic properties to the previously characterized promiscuous *Nicotiana tabacum* double bond reductase NtDBR (33). For example, both RZS1 and NtDBR share increased activity towards acidic pH conditions (Figure S4). For all further RZS1 enzyme assays, these were measured at 30°C and pH 6.4, with 1 mM of phenylbuten-2-one. Under these conditions, RZS1 shows an apparent *K*_cat_/*K*_m_ of 58- and 1.3 mM s^−1^ mM^−1^ with NADPH and NADH, respectively (Table S3).

**Figure 2.**
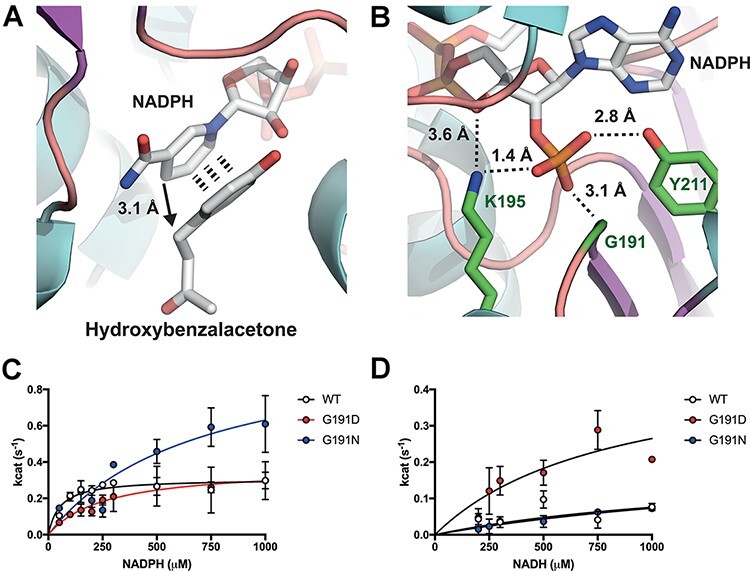
Structure based engineering of the RZS1 reductase with increased NADH specificity (pdb: 6EOW). A. Binding site for HBA in proximity to the NADPH cofactor. B. Cofactor specificity is provided by a triad of binding residues G191, K195 and Y211 with hydrogen bonding to the 5ʹ-ribose phosphate. C. NADPH kinetics with RZS1 G191 variants. D. NADH kinetics with RZS1 G191 variants.

About 10% of all known oxidoreductase enzymes, particularly from secondary metabolism, have a distinct preference for NADPH (34). A number of studies have also highlighted that the cofactor specificity of NADPH-dependent reductase enzymes can be relaxed towards NADH preference by increasing the interaction of the enzyme with the ribose 5ʹ-hydroxyl group (NADH) (1, 27). To aid in the rational design of increasing NADH activity of the RZS1 enzyme, we crystallized and solved the tertiary structure complex of RZS1 with raspberry ketone pathway substrate HBA (Figure 2A) and the cofactor NADPH (Figure 2B), deposited as pdb:6EOW. By analyzing the 2*F_0_*—*F_c_* Fourier syntheses, two configuration states were observed in a 50:50 ratio due to crystal soaking. The mixed states could be as a result of NADPH and ternary complex formation to the active site, thus displacing any NADP^+^ present from crystallization. In the ternary complex state, additional electron density for HBA binding is observed, with face-to-face π-π stacking between the HBA and nicotinamide aromatic rings (Figure S5) with a hydride transfer distance of 3.06 Å to the alkene double bond. Furthermore, in the ternary complex, an additional patch of electron density consistent of a flexible loop supporting Y72 is observed. This flexible loop forms a cap over the active site (closed loop), with the *para* hydroxyl group in HBA moving towards Y72. With respect to HBA binding, the substrate is bent within the active site and is encased by aromatic residues Y59, Y72, Y85, F107, Y263 and F290 (Figure S5). We speculate that this closed loop formation holds the HBA within the active site prior to transition state. Interestingly, in contrast to apo-RZS1 and NtDBR, the Y72 flexible loop lacks electron density and seemingly points away from the active site (open loop state). Finally, in regard to cofactor binding, in both states the NADP(H/^+^) cofactor is bound in a typical conformation as is observed in the previous related structures (33) with the binding specificity provided by a triad of hydrogen bond based contacts with the G191 backbone nitrogen and the side chains K195 and Y211 (Figure S5). While K212 is also present, it is rather removed from binding to the ribose 5ʹ-phosphate. Importantly, K195 can interact with either the ribose 5ʹ-phosphate or neighboring 4ʹ-hydroxyl group.

In an effort to modify the cofactor specificity of the RZS1 enzyme, amino acid substitutions at G191 (Table S3) and Y211 (data not shown), and steady-state kinetics was determined (Figure 2 and Table S3). We did not attempt to modify K195 due to its potential role in bonding to the 4ʹ hydroxyl group found in both NADPH and NADH. We instead chose to modify G191 to provide a short polar side chain (D and N) that can provide a hydrogen bond for the 5ʹ-hydroxyl group unique to NADH. Both the D191 and N191 modifications decreased the *k*_cat_/*K*_m_ from 58 mM^−1^ s^−1^ (RZS^WT^) with NADPH to 14 and 15 (s^−1^ mM^−1^), respectively (Figure 2C and D). This decrease in catalytic efficiency was due to an increased *K*_m_ for NADPH with the variants. Interestingly, for N191, the *k*_cat_ increased by 3-fold up to 1.07 s^−1^ in comparison to the wild-type (0.31 s^−1^). One possible interpretation is that steric hindrance of N191 with the 5ʹ phosphate increases the catalytic rate by enhancing NADP^+^ release through decreasing its binding affinity. In contrast, with NADH as the cofactor, while N191 did not improve NADH specificity, D191 was found to favorably increase the *k*_cat_/*K*_m_ from 1.3 mM^−1^ s^−1^ (RZS^WT^) to 5.9 mM^−1^ s^−1^ (Figure 2 and Table S3). In summary, substitutions at G191 that are negatively charged (D) improve NADH specificity in preference to a positive charged residue (N). While further modifications to the Y211 position were also tested individually or in combination with D191 and N191 (data not shown), no further improvements in NADH specificity or *k*_cat_ were found, with modifications leading to a reduction in specific activity with either NADH or NADPH.

### **4.** **3** NADH based enzymatic synthesis of raspberry ketone from HBA

**Figure 3.**
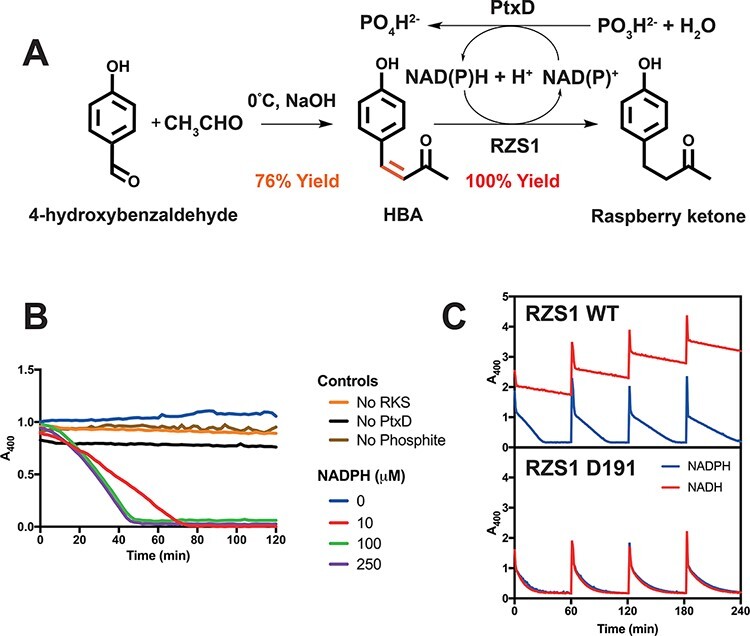
A two-step semi-synthetic route to high-yield raspberry ketone synthesis with NADH and cofactor regeneration. A. A semi-synthetic pathway for raspberry ketone using aldol condensation and RZS1 reductase activity with cofactor regeneration. B. Activity of the thermostable PtxD opt12 phosphite dehydrogenase with 1 mM HBA and 10 μM RZS1. Negative controls (no enzyme or phosphite) are shown along with a variable concentration of NADPH. C. Time-course reaction monitoring loss of absorbance at 400 nm showing reduction of HBA to raspberry ketone. Injections of 1 mM HBA were added in 60 min cycles. An excess of PtxD and 20 mM phosphite was incubated at 30°C with 10 μM RZS1 (top panel) or the D191 variant (bottom panel), with either 0.25 μM NADPH (blue line) or NADH (red line).

The kinetics of RZS1 (wild-type and D191) were not characterized with its natural substrate, HBA. Instead, we studied its activity with HBA and NAD(P)H cofactor regeneration in a ‘one-pot’ reaction. First, HBA was synthesized from the inexpensive substrates *p*-benzaldehyde and acetic acid using an aldol condensation reaction under basic conditions to provide a 76% yield (Figures 3A and S6). Then, using the thermostable phosphite dehydrogenase (PtxD) mutant opt12 (35) for NAD(P)H regeneration from phosphite, we tested the reduction of the HBA substrate at 30°C by following a loss of absorbance at 400 nm (A400). For negative controls, in the absence of the reductase, phosphite or PtxD, the A400 for 1 mM HBA remained stable over the time-course measured (Figure 3B). With 1 mM HBA, 10 μM of RZS1, 20 mM phosphite and an excess of PtxD opt12, complete reduction was achieved with as little as 10 μM of NADPH (Figure 3). Next, to demonstrate the proficiency of the RZS1 variants (10 μM) with both NADPH and NADH, a time-course reaction was monitored with 1 mM injections of HBA every hour with 0.25 mM NAD(P)H, 20 mM phosphite and an excess of PtxD opt12 (Figure 3C). For RZS1^WT^, an initial rate of 59.1 and 12.8 μM min^−1^ mg^−1^ was observed with NADPH and NADH, respectively (Table S2). In comparison, for RZS1^D191^, the rates of reduction with NADPH and NADH were nearly equivalent at 46.5 and 43.5 min^−1^ mg^−1^, respectively (Table S2). This demonstrated highly efficient synthesis of raspberry ketone, with the RZS^D191^ variant providing an elevated and complete turnover (∼100%) with the inexpensive NADH cofactor. We also found that RZS1 reductase activity lasted for several days at room temperature, which is a desirable property for cell-free enzyme biosynthesis (data not shown).

### **4.** **4** NADH based ‘one-pot’ biosynthesis of raspberry ketone from L-tyrosine

**Figure 4.**
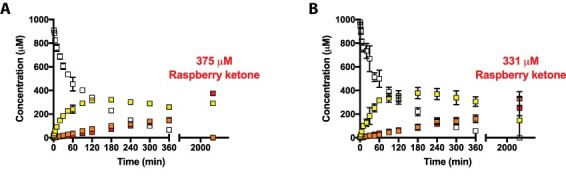
‘One-pot’ biosynthesis of raspberry ketone under optimized enzyme levels and cofactor regeneration. A Reaction with RZS1^WT^ and NADPH. B Reaction with RZS1^D191^ and NADH. Reaction conditions are provided in Table S2.

To understand whether the RZS1^D191^ variant could be used for cell-free synthesis of raspberry ketone with NADH, three time-course reactions were prepared with 1 mM L-tyrosine and optimized enzyme levels and cofactors with either an absence of the reductase or a combination of RZS1^WT^/NADPH or RZS1^D191^/NADH. Without RZS1, the reaction accumulated HBA at a rate of 0.81 μM min^−1^ (Table S2), with a maximum conversion of 421 μM observed after 48 h, along with 197 μM of leftover *p*-coumarate. By adding 20 μM RZS1^WT^ with 0.5 mM NADPH along with cofactor recycling with the phosphite dehydrogenase (35), in contrast, a linear rate of raspberry ketone synthesis of 0.38 μM min^−1^ was observed. After 48 h, the final concentrations of the intermediates were 291 μM *p*-coumarate and 375 μM raspberry ketone, while no L-tyrosine or HBA was detected (Figure 4). The HPLC chromatogram trace suggested that the remaining mixture was composed of the bisnoryangonin side-product, previously shown as a minor side-product by the BAS enzyme at low pH (36). In contrast, with 20 μM RZS1^D191^ introduced under the same conditions, the final level of raspberry ketone synthesis was improved to 279 μM at a rate of 0.51 μM min^−1^, with a mixture of 297 μM *p*-coumarate and 198 μM HBA remaining (Figure 4).

## 5. Discussion

The related areas of cell-free synthetic biology and synthetic biochemistry offer an exciting opportunity to design and engineer enzyme pathways outside of the constraints of a living cell. Recent advances include the generation of a synthetic CO_2_ fixation cycle (37) and the 20 000 L scale-up biosynthesis of inositol (38). Natural products and other high-value fine chemicals are often only synthesized at very low concentrations in their natural source. Therefore, there is an accelerating interest in developing biosustainable routes for a range of high-value fine chemicals. For raspberry ketone, only tiny quantities are synthesized in the berries themselves and the process requires weeks of maturation. While the raspberry ketone intermediates in general inhibit microbial growth production, this can be bypassed using a *E. coli* whole-cell (stationary phase cells) biocatalyst system which is active to the g L^−1^ scale but requires the chemically derived precursor HBA (24). Alternatively, the substrates L-tyrosine or *p*-coumarate, which can be derived from a natural feedstock, can be converted by the type III polyketide described herein, in synthetically engineered *E. coli* and *S. cerevisiae* systems but yields are limited (up to 95 mg L^−1^) due to general toxicity (15, 21, 22).

Herein, we have investigated an alternative cell-free ‘one-pot’ multienzyme system for the biosynthesis of raspberry ketone. Raspberry ketone, although one of the simpler polyketides, still has a biosynthetic pathway that requires the expensive cofactors ATP, malonyl-CoA, and NAD(P)H. By studying this system outside of the cell, this allows the precise control of enzyme levels, and flexible testing of different cofactor recycling systems. We demonstrate that raspberry ketone can be synthesized efficiently from a five-step enzyme pathway from L-tyrosine, however, yields with the most cost-effective cofactor, NADH, were limited. Therefore, to reduce a key cost-factor, NADPH, a key focus of our work, was to relax the cofactor specificity of the RZS1 double bond reductase towards NADH. A number of studies have highlighted that NADPH oxidoreductases can be engineered to use the more stable and inexpensive NADH (27) or biomimetic analogues (32). As a generalized approach, the structure-guided design of NAD(P)H enzymes can be rationalized by altering the affinity for the ribose 5ʹ-phosphate (NADPH) or hydroxyl (NADH) group (27). In the case of the RZS1 enzyme, position G191 provides flexible control for engineering relaxed cofactor specificity. Its coupled use with an inexpensive phosphate donor via the phosphite dehydrogenase (35) thus affords a low-cost route to raspberry ketone synthesis in cell-free conditions with NADH using any of the three starting substrates described. Importantly, the RZS1 G191D variant is efficient at low levels of NADH (10 μM) and remains active for several days. By substituting this enzyme into the complete ‘one-pot’ multienzyme system, we can obtain a maximum yield of 61 mg L^−1^ raspberry ketone in a batch system with full conversion of the substrate L-L-tyrosine (300 μM or 54 mg L^−1^) into raspberry ketone. At higher concentrations of L-tyrosine, despite attempts to optimize cofactor and ATP levels (data not shown), we observe accumulation of pathway intermediates and the side-product bisnoryangonin. Finally, we also demonstrate an efficient two-step chemoenzymatic approach to raspberry ketone. This is similar to the strategy employed by Yang *et al* (23), which reaches a product that is ‘nature identical’ and of lower economic value, due to the requirement to chemically synthesize HBA. Therefore, to make natural raspberry ketone, the five-step pathway is of greater interest if scalability can be demonstrated. However, either further engineering of the BAS enzyme, the characterization of new BAS homologs, or the development of alternative enzyme pathways is required. This is since the catalytic activity of BAS is rate-limiting and it also produces an undesired side-product, bisnoryangonin, in our optimized reactions.

While a full technoeconomic analysis is outside the scope of the current work, a cell-free enzymatic approach to fine chemical synthesis could provide benefits in terms of sustainability. Approximately 80% of the fine chemical market that is used for cosmetics and food additives, is currently produced by oil derived chemical synthesis and thus approved for use if declared as ‘nature identical’(39). For fine chemicals that are extracted naturally from food sources that require large agricultural landmasses, therefore, potentially it is far simpler and more sustainable to engineer greener alternative biocatalytic platforms, either from the use of engineered plants and microbes or through scaled-up cell-free systems. The use of purified enzymes compared to whole-cell processes for production might offer benefits in terms of control over side reactions, ease of purification because of fewer contaminating metabolites, or for situations where the toxicity of the product limits cell growth. However, the costs of enzyme production and purification, along with the need to add expensive cofactors that would normally be generated from medium components within cells does lead to increased costs. Strategies such as immobilization of the enzymes to enable recycle and reuse, could potentially be employed to achieve economic viability (40).

Here, using raspberry ketone as a model pathway, we have demonstrated how an apparently non-productive enzyme pathway can be engineered to a high level of performance from outside of the cell. Essential to this process is the ability to study enzyme pathways in completion, rather than as individual uncoupled kinetics, since shared resources (CoA, ATP) can impact overall yields. In summary, cell-free synthetic biology provides an expandable opportunity to design synthetic enzyme ensembles in unison with cofactor availability. We have applied this rational to optimize raspberry ketone, a small molecule that is not easily obtained from within engineered living cells. In the future, scaled-up cell-free engineering approaches (38) with highly stable and active enzymes, have the potential to play a broader role to compete with traditional cell-based engineering, particularly for the high-value fine chemicals, recombinant proteins and natural products (5).

## Supplementary Material

ysab021_Supp

## Data Availability

Plasmids are available at reasonable request from Imperial College London via a Material Transfer Agreement.
